# Editorial: Genetics and epigenetics in ovarian aging

**DOI:** 10.3389/fendo.2025.1555914

**Published:** 2025-02-11

**Authors:** Honey V. Reddi

**Affiliations:** Belay Diagnostics, Chicago, IL, United States

**Keywords:** ovarian aging, genetics, epigenetics, mitochondria, endocrinology

Reproductive aging begins in a woman’s mid-30s, with menopause usually occurring between the ages of 48 and 50, and the depletion of the oocyte stock (ovarian aging) being an inevitable process during a woman’s lifetime that ultimately influences life expectancy and health outcomes. Ovarian aging is a multidimensional process characterized by a progressive decline in follicle number and oocyte quality that accelerates around approximately 37 years of age, leading to increased infertility and congenital disabilities in offspring (1). Despite its importance, little is known about the basic biological mechanisms that underlie human ovarian aging, particularly in terms of prolonging female fertility and improving population quality.

While human life expectancy has significantly extended over the past century, menopausal age has remained largely unchanged suggesting an underlying role for genetic and epigenetic factors, Perimenopause marks the initiation of female reproductive senescence, with the age of onset being observed to be heritable in only 47% of cases (2), suggesting that additional factors other than genetics such as age at menarche, oral contraceptive use, alcohol consumption, smoking, and level of physical activity (3, 4) regulate this endocrine aging transition. Recently, aging of the hypothalamic-pituitary axis along with diminished telomerase activity has gained recognition as a critical catalyst for reproductive aging (5). Follicular atresia due to apoptosis in the granulosa and oocytes triggered by excessive production of reactive oxygen species (ROS) also contributes to ovarian aging.

Recent studies have identified genetic polymorphisms as a major contributor to the heterogeneity in natural menopause age, especially for genes involved in the DNA repair pathway. Pathological ovarian aging, such as premature ovarian insufficiency and early menopause, has also demonstrated similar genetic susceptibility (6). Central to this phenomenon are epigenetic modifications in follicular development and maintenance of ovarian function, particularly DNA methylation, which exert a significant influence on gene expression during pivotal stages of ovarian development. These studies offer the opportunity to elucidate the influence of the interaction between genetics and environment on ovarian aging. This Research Topic highlights some of the advances made in delineating the genetic and epigenetic mechanisms of physiological and pathological ovarian aging, providing insights into potential mechanisms to extend the female reproductive lifespan.

Research indicates a connection between DNA methylation (DNAm) aging and reproductive aging; however, the causal relationship between DNAm and age at menopause remains uncertain. Technological advancements have made it possible to measure biological age using various molecular or phenotypic biomarkers. Wang L. et al. in their study showed that an increase in granulocyte DNAm levels in relation to menopausal age could potentially serve as a valuable indicator to evaluate physiological status at the onset of menopause.

Diminished ovarian reserve (DOR) refers to a decrease in the number or quality of oocytes in the ovarian cortex, which is a degenerative disease of the reproductive system, and can further develop into premature ovarian failure. Zhang et al. in their study provided clinical evidence and theoretical support for the treatment of DOR with conception vessel acupuncture and moxibustion. Similarly, in evaluating therapeutic options to boost oocyte quality, Zhao et al. observed that daily oral intake of L-carnitine before oocyte retrieval could boost oocyte quality and embryo development, thus improving IVF outcomes. They also suggested that ongoing investigations have the potential to offer valuable insights into the applications and mechanisms underlying the therapeutic effectiveness of L-carnitine. The study investigated the relationship between L-carnitine treatment and *in vivo* oocyte maturation, normal fertilization, and subsequent embryo development using a total of 515 *In Vitro* Fertilization (IVF) patients undergoing subsequent cycles who were included after applying exclusion criteria.

Recent studies have identified mitochondria as pivotal players in ovarian aging, influencing various hallmarks and pathways that govern this intricate process (PMID: 27562289). Wang Z. et al. in their review provided a deeper understanding of the intricate interplay between mitochondrial function and ovarian aging, offering valuable perspectives for the development of novel therapeutic interventions. The authors explored the potential of targeting mitochondrial dysfunction through innovative therapeutic approaches, such as antioxidants, metabolic improvement, biogenesis promotion, enhanced mitophagy, mitochondrial replacement therapy (MRT) and traditional Chinese medicine (TCM) treatments. They summarize the current major mitochondrial therapies in Table 1 (Wang Z. et al.). These therapies show great potential in alleviating ovarian aging, however are still facing some challenges and limitations in practical application. Similarly, Mani et al. provided information on the latest insights into the mechanisms involved in ovarian aging and the possibility of preserving ovarian function leading to the extension of ovarian longevity. They described the role of mitochondria-led epigenetics in ovarian aging and discussed strategies to restore epigenetic reprogramming in oocytes by preserving, protecting, or promoting mitochondrial function. The authors also presented evidence to demonstrate that nuclear and mitochondrial genomes cross-talk with each other, resulting in a two-way orchestrated anterograde and retrograde response that involves epigenetic changes in nuclear and mitochondrial compartments. These epigenetic alterations, which cause changes in metabolism impact ovarian function, so enhancing mitochondrial function in aging ovaries may preserve ovarian function and may lead to ovarian longevity and better reproductive and health outcomes in women.

In conclusion, this Research Topic on ‘*Genetics and Epigenetics in Ovarian Aging*” showcases contributions to the field that focus on the potential biological mechanisms underlying human ovarian aging, particularly with regard to prolonging female fertility and improving population quality. They explore the potential of targeting mitochondrial dysfunction through innovative therapeutic approaches, that could ultimately preserve ovarian function, extending the female reproductive lifespan. We thank the contributing authors for their efforts and hope that their studies will be appreciated by the readers.
